# Can oxidative potential be a plant risk indicator for heavy metals contaminated soil? Analysis of ryegrass (*Lolium perenne* L.) metabolome based on machine learning

**DOI:** 10.1016/j.eehl.2025.100140

**Published:** 2025-03-03

**Authors:** Chunmei Ran, Meiqi Guo, Yuan Wang, Ye Li, Jiao Wang, Yinqing Zhang, Chunguang Liu, Bridget A. Bergquist, Chu Peng

**Affiliations:** a Department of Earth Sciences, University of Toronto, Toronto, Ontario M5S 3B1, Canada; b MOE Key Laboratory of Pollution Processes and Environmental Criteria, College of Environmental Science and Engineering, Nankai University, Tianjin 300071, China; c School of Energy and Environmental Engineering, Hebei University of Technology, Tianjin 300401, China

**Keywords:** Oxidative potential, Heavy metal contaminated soil, Plant health, Metabolomics, Machine learning

## Abstract

Evaluating the plant risk of soil pollution by plant physiological indices usually requires a long cycle and has significant uncertainty. In this study, oxidative potential (OP) of the *in situ* heavy metal contaminated soils was measured by the dithiothreitol method. The oxidative stress response of the model plant ryegrass (*Lolium perenne* L.) induced by heavy metal contaminated soil was evaluated by the biomarkers, including superoxide dismutase and total antioxidant capacity. The comprehensive biomarker response index has a significant exponential correlation with the OP of soil (*r* ​= ​0.923, *p* ​< ​0.01) in ryegrass. Metabolomics analysis also showed a significant relationship of the metabolic effect level index of amino acids and sugars with OP. Random forest was selected from four machine learning models to screen the metabolites most relevant to OP, and Shapley additive explanations analysis was used to explain the contribution and the influence direction of the features on the model. Based on the selected 20 metabolites, the metabolic pathways most related to OP in plants, including alkaloid synthesis and amino acids metabolism, were identified. Compared to the plant physiological indices, OP is a more stable and faster indicator for the plant risk assessment of heavy metals contaminated soil.

## Highlights

• Oxidative potential (OP) is exponential correlated with the comprehensive biomarker response index.
• OP was significantly related to the metabolic effect level indexes of amino acids and sugars.
• Top 20 important metabolites in ryegrass contributed to OP was screened based on random forest.
• The metabolic pathways in ryegrass most related to OP were identified.

## Graphical abstract

**Figure:**
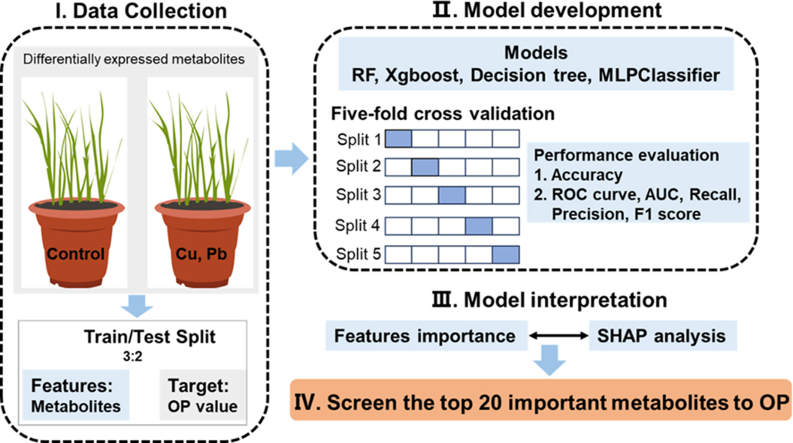

## 1 Introduction

Heavy metal-contaminated soil poses widespread health risks to plants [1]. Cu and Pb are major soil contaminants widely distributed in industrial sites and can disrupt the redox state of plant cells by generating reactive oxygen species (ROS) [2], which is considered a crucial mechanism of the ecological risk posed by Cu and Pb. ROS can negatively affect important physiological processes such as plant respiration and photosynthesis, ultimately leading to toxicity symptoms such as growth retardation, root blackening, and yellowing in plants [3,4]. For example, ROS can result in metabolic imbalances in plant cells [5], affect membrane structure and permeability, and inhibit photosynthesis of plants [6]. Ryegrass (*Lolium perenne* L.) is a widely distributed, fast-growing plant with high biomass. It plays a vital role in maintaining the functions of soil ecosystems and has the ability to tolerate soil contamination, such as heavy metals. In addition, the growth status and physiological indicators of ryegrass are sensitive to heavy metals, so it can serve as a model plant for evaluating the ecological risk of heavy metals contamination [7,8]. The oxidative stress of heavy metals on plants was usually measured by indicators such as antioxidant enzymes and malondialdehyde (MDA). However, the indicators have limitations, such as the long time required for plant cultivation and multiple factors that can cause increased bias in evaluation, including interspecific or individual variations of plants and environmental factors like soil properties and temperature [9].

Oxidative potential (OP) is an index reflecting the integrated contents of redox-active components in contaminated particle matters [10]. Dithiothreitol (DTT) assay can quantitatively measure particle matter-mediated ROS formation, and Cu and Pb have been proven to be contributors to OP values measured by the DTT method. In addition, OP value has been found to be associated with several biological endpoints, such as congestive heart failure, lung cancer, and asthma in humans [[11], [12], [13], [14]] and antioxidant enzymes in soil animals [15,16], indicating that OP may be a reasonable predictor parameter for ROS induced oxidative stress effects [12,17,18]. Although there are differences in the oxidative damage of heavy metals to animals and plants [19,20], OP is also speculated to be applicable for assessing ecological risks caused by ROS induced by heavy metals to plants and may have a dose–response correlation with oxidative stress indicators in plants. However, there is still no direct evidence for the correlation between soil OP and oxidative stress indicators in plants, such as antioxidant enzymes and metabolomics, which limits the application of OP in ecological risk assessment of heavy metal-contaminated soils to plants. Meanwhile, due to the large scale and interrelationship of metabolites, traditional mathematical methods are unable to screen out the most important metabolites related to OP as a whole, and the accuracy of the screening is limited.

Machine learning has the ability to effectively select the most important features because of the classification function, and is suitable for complex omics data analysis due to the high flexibility of its algorithms [21]. Tree-based algorithms, such as extreme gradient boosting (XGBoost), decision tree and random forest, and neural networks, such as multi-layer perceptron classifier (MLPClassifier), are common modeling methods used in machine learning [22]. These algorithms can provide insights into the potential interactions between numerous input features and output results, thereby improving the prediction accuracy [23]. Machine learning has been applied in biological models and has been proven to be effective in genomic selection, treatment target prediction, and drug target prediction [24,25]. Technically, metabolites related to OP can be ranked and selected, and the accuracy of screening can be improved based on machine learning.

To further explain the results of machine learning screening, explainable machine learning was used for analysis. As a new branch of machine learning, explainable machine learning can better explain the prediction results of machine learning models [26]. It provides details such as the correctness, robustness, bias, improvement, and transferability, and further reveals the dependencies between inputs and outputs and the importance of inputs [27]. Shapley additive explanations (SHAP) and local interpretable model-agnostic explanations (LIME) are the common explainable machine learning techniques. LIME weights instances based on their similarity to the corresponding original instances, while Shapley values in SHAP analysis are calculated by estimating the weights corresponding to sampled instances. Compared to LIME, SHAP can provide a global explanation by combining individual local explanations and using predictions with a fair distribution among the input [28]. SHAP value is considered a unified measure of feature importance, and SHAP has an advantage over LIME. Therefore, SHAP was selected to explain the feature contribution and the direction of feature influence on the model.

To assess the applicability of OP in evaluating the ecological risk of heavy metal-contaminated soil on plants, the potential relationship between soil OP values and the oxidative stress indicators in ryegrass *Lolium perenne* L.) planted in heavy metals contaminated soils was analyzed. Using machine learning, the most relevant metabolic perturbations associated with OP were screened from metabolomics and explained by SHAP analysis to prove that OP can be used as a rapid and stable indicator for assessing risks in ryegrass under oxidative stress caused by heavy metals.

## 2 Materials and methods

### 2.1 Chemicals and soils

The reagents for OP determination, including DTT, phosphate-buffered saline (PBS) buffer (pH 7.4), and 5,5-dithiobis(2-nitrobenzoic acid) (DTNB) and the reagents for heavy metal determination, including HNO_3_ and HF, were purchased from Sigma Aldrich (Shanghai, China) and Aladdin Reagent Co., Ltd. of China, respectively. Fisher Scientific Inc. (Loughborough, UK) provided acetonitrile of chromatographic grade, ammonium acetate, ethanoic acid, and methanol. All the chemicals purchased were of the highest purity available. The total protein, MDA, superoxide dismutase (SOD), hydrogen peroxide (H_2_O_2_), peroxidase (POD), metallothionein (MT), catalase (CAT), and total antioxidant capacity (T-AOC) assay kits were bought from Nanjing Jiancheng Bioengineering Institute (Nanjing, China).

A metal smelter plant (Shenyang, Liaoning Province, China) provided the topsoil samples contaminated with Cu and Pb (i.e., S1–S8), and the concentrations of Cu and Pb in the soil are shown in Table S1. The soil located 3 ​km upwind from the plant was selected as the control. The physical and chemical properties of soil are shown in Table S2, and the determination is described in a previous study [15]. The soils were air-dried and sieved to a particle size of <2 ​mm for ryegrass cultivation and sieved to <0.15 ​mm for Cu and Pb concentrations detection and OP determination. Cu and Pb concentrations were analyzed using inductively coupled plasma mass spectrometry (ICP-MS) (Elan DRC-e, PerkinElmer, Waltham, MA, USA) after soil digestion, and the digestion procedure was described in previous literature [29]. The limit of detection for ICP-MS was 0.5 ​μg/L. The OP of soil water extracts was assessed by the DTT assay, as described in previous research [15].

### 2.2 Ryegrass exposure design

Ryegrass (*Lolium perenne* L.) seeds were obtained from a local gardening supply store. The ryegrass seed surfaces were sterilized with a solution of 3% H_2_O_2_ for a duration of 20 ​min, and then sowed in the blank soil for 14 days. When the ryegrass reached a height of about 15–20 ​cm, 20 ryegrass plants with similar growth were selected and transplanted into the pots filled with 2.5 ​kg of control soil or S1–S8 contaminated soils for 21 days [30]. The pots were incubated at 20 ​± ​1 ​°C and light cycle of 16 ​h/8 ​h (day/night). Ryegrass leaf samples were cleaned with PBS and collected after 21 days for biomarkers and metabolomics determination.

### 2.3 Biomarkers assay

Fresh leaf tissues were ground using liquid nitrogen mixed with PBS solution, followed by centrifugation at 4 ​°C for 15 ​min at 5,000×*g* to extract the supernatants. Supernatants were collected and used for subsequent determination. Details on the assessment of total protein, ROS, MDA, T-AOC, MT, as well as the activities of CAT, POD, and SOD and the calculation of biomarker response index (BRI) has been described in the previous research [12,15,31,32].

### 2.4 Metabolomics detection and profiling

Ryegrass leaves exposed to S1, S2, S5, S6, and the control groups were used for metabolomics analysis. Samples with 25 ​mg were extracted using a 500 ​μL mixture of acetonitrile:methanol:water in a 1:2:1 ratio with isotopic internal standard. Subsequently, samples were homogenized at 35 ​Hz for 4 ​min and sonicated in an ice water bath for 5 ​min and repeated three times. The samples were then incubated at −40 ​°C for 1 ​h and centrifuged at 13,800 × *g* at 4 ​°C for 15 ​min. The obtained supernatants were used for analysis. The mixture of an equal amount of supernatant from all samples was prepared for the quality control sample [33,34]. The masses of the metabolites were analyzed by LC-MS/MS [35,36]. The LC gradient elution programs and mass detection are detailed in Table S3. Metabolic analysis, including metabolite identification and pathway enrichment, is described in the previous research [15].

### 2.5 The calculation of the metabolic effect level index

The metabolic effect level index (MELI) is a quantitative endpoint to reflect the aggregated response of the changes in all individual metabolites after exposure [37].

Firstly, the metabolic changes (MC) for each individual metabolites in plants samples of the contaminated group compared to the average MC in plants of the control group were calculate as Eq. 1:

(1) $${MC}_{i,T}=e^{\left|\ln A_{i,T}\right|}-e^{\left|\ln A_{i,C}\right|}$$

Here *A*_*T*_ is the ratio of the abundance of individual metabolite *i* in the contaminated group to the mean abundance of these metabolites in the control group, and *A*_*C*_ is the relative metabolic change of the control group samples, which, by definition, equals to 1. Then MELI was calculated as Eq. 2, dividing the sum of MC for all *n* metabolites in the sample by the number of metabolites *n*:

(2) $$MELI={MC}_{total,T/n}=\sum_{i=1}^{i=n}{MC}_{i,T}/n$$

In this study, MELI for different classes of metabolites was calculated separately.

### 2.6 Machine learning for identifying important metabolites related to OP

#### 2.6.1 Machine learning models

In order to further identify metabolites related to OP, the machine learning analysis based on the results of differential expression analysis was conducted. The whole process of machine learning analysis is illustrated in Fig. S1. To find the machine learning model that best fits the data in this study, four commonly used classification models, including XGBoost, MLPClassifier, decision tree, and random forest, were tested [21]. A brief introduction to the principles of four machine learning models is described in Text S1, and the details of the algorithms can be found in previous literature [22,28,38,39]. For the purposes of the model performance evaluation, each of the machine learning models was applied to the same normalized data set. Significantly differential metabolites in S1, S2, S5, and S6 groups compared to the control group were selected and used for model analysis. The data set was divided into training (60%) and test (40%) sets prior to the application of machine learning [21].

#### 2.6.2 Model optimization and performance evaluation

The grid search method was used for hyperparameter optimization of the four machine learning models [28]. The grid search method evaluates the models by selecting the most appropriate set of hyperparameters. The optimized models were then used for validation purposes.

Metrics for evaluating model performance, including cross-validation accuracy, recall, precision, F1 score, receiver operating characteristic (ROC) curves and the area under the ROC curve (AUC), and the calculation of cross-validation accuracy, recall, precision, and F1 score are described in Text S2. Five-fold cross-validation was used to evaluate the accuracy of the models. Based on the metrics, the best model was selected, and all metabolites with differential expression compared to the control group were ranked using the selected model to identify the top 20 important metabolites related to OP values.

#### 2.6.3 SHAP analysis

SHAP analysis interprets machine learning models through SHAP values [27]. A brief introduction to the principles of SHAP is described in Text S3, and the details of the algorithm can be found in previous literature [26,28]. Each feature possesses a SHAP value representing its average marginal contribution. A higher absolute SHAP value indicates a greater influence of the feature on the model, with positive or negative values indicating the average impact on the model's prediction [40]. Features that have a significant impact on the model output can be identified through SHAP global explanations, which rank the features based on the SHAP values.

### 2.7 Statistical analysis

SPSS software (V22.0, Chicago, USA) was used to identify significant differences through one-way analysis of variance (ANOVA, *p* ​< ​0.05) and post hoc testing using Fisher's least significant difference (LSD). Additionally, the R software V.3.3.5 package Ropls was used for univariate (Student's t-test) testing and multivariate analyses, including principal component analysis (PCA). The machine learning model was developed using the package Scikit-learn V.1.4 and SHAP V.0.45.1. All steps were performed using Python V.3.12.2 and Jupyter Notebook. Pandas V.2.2 and NumPy V.1.26.3 were used for basic statistical calculations and data collation, respectively.

## 3 Results and discussion

### 3.1 OP values of heavy metals contaminated soil

OP values were measured by the DTT oxidation efficiency. The OP of the control group was much lower than that of the contaminated groups, with values of 20.9 and 25.5–62.8 pmol/(min·μg), respectively (Table S1). Cu and Pb concentrations in water extracts of contaminated soils were 0.155–1.93 ​and 0.00399–0.384 ​μM, respectively, while the Cu concentration in water extract of control soil was 0.0783 ​μM, and Pb was lower than the detection limit. The OP values increased with the increase of total Cu and Pb concentration in water extracts (Table S1).

Heavy metals could be the dominant species responsible for catalyzing DTT oxidation [15]. Transition metals such as Cu and Pb play a dominant role in ROS formation from the soil samples. The family of ROS, including O_2_^·–^, HO_2_, H_2_O_2_, and ·OH, is linked by multiple reactions beginning with O_2_ accepting an electron from DTT catalyzed by Cu and Pb to become O_2_^·–^. O_2_^·–^ can add H^+^ to form HO_2_, which leads to the formation of H_2_O_2_ and then ·OH, which is eventually converted to H_2_O [41]. Transition metals with particle matter-induced cytotoxicity and health effects have been reported [42] and explained by the formation of ROS [43].

### 3.2 Oxidative stress on ryegrass and correlation with OP

**Fig. 1:**
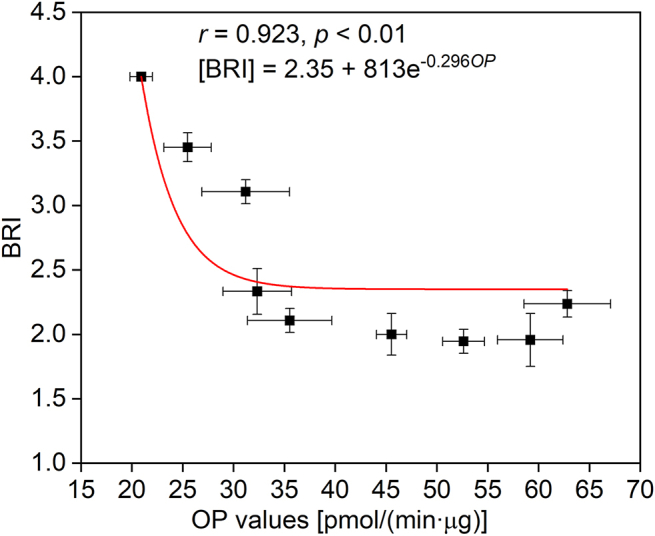
Correlation between OP values of the soils and biomarker response index (BRI) of the ryegrass (*Lolium perenne* L.*).*

Heavy metals can severely damage the defense system by disturbing the normal metabolic processes in plants, ultimately affecting the production of antioxidants in plants [44]. The increase in ROS levels in plants compared to the control group also indicated that heavy metals induced oxidative stress in ryegrass (Fig. S2). The activity of biomarkers, including antioxidant enzymes (i.e., SOD, CAT, POD), T-AOC, MDA, and MT, in the ryegrass leaves under the Cu or Pb stress changed significantly compared to the control group (Fig. S3). T-AOC, MT, and POD in leaves of the contaminated group were significantly higher than those in the control groups, while SOD, CAT, and MDA were significantly lower than those in the control group (*p* ​< ​0.05). Antioxidant enzymes (e.g., SOD, CAT, POD), assessment indicators of oxidative level (e.g., T-AOC, MDA), and antioxidant molecules (e.g., MT) are a common plant response to oxidative stress. Due to the significant differences in dose–response relationships among different biomarker responses, BRI, as an integrated index based on multiple biomarker responses, was used to establish dose-effect relationships between contamination levels and biomarker responses [45]. In this study, BRI varied from 1.95 to 3.45 for ryegrasses exposed to industrial-contaminated soils (Fig. 1), while BRI of the ryegrass exposed to the control soil was 4.00, indicating that heavy metals caused slight or severe changes of the physiological status of ryegrass, respectively [45].

BRI has a significant exponential correlation with OP (*r* ​= ​0.923, *p* ​< ​0.01) (Fig. 1), and OP values have been linked to the health effect indicators of particles, such as airway inflammation markers [46], cellular cytotoxicity [47,48], cellular oxidative stress markers [49], and cardiorespiratory health endpoints [12,14]. Although there is a good exponential relationship between BRI in earthworms after 28 days of exposure and the concentration of antimony [50], for the first time, OP was linked to the biomarker changes (i.e., BRI) in plants, an indicator of oxidative stress induced by heavy metals. The exponential relationship between OP and BRI also indicated that the range of BRI variance at lower OP values was greater than that at higher OP values. This may be because plants can self-regulate through their own antioxidant systems to slow down cell damage and resist oxidative stress, while higher concentrations of heavy metals (i.e., at higher OP values) can quickly induce plant tolerance mechanisms, leading to inconsistent changes in biomarkers and OP values [51]. As a whole, the OP values can be used as an index for assessing the oxidative stress from lower heavy metal contamination in soil on plants.

### 3.3 Relationship between OP values and metabolomics response of ryegrass

#### 3.3.1 Changes in metabolic profiles of ryegrass exposed to soils with different OP

**Fig. 2:**
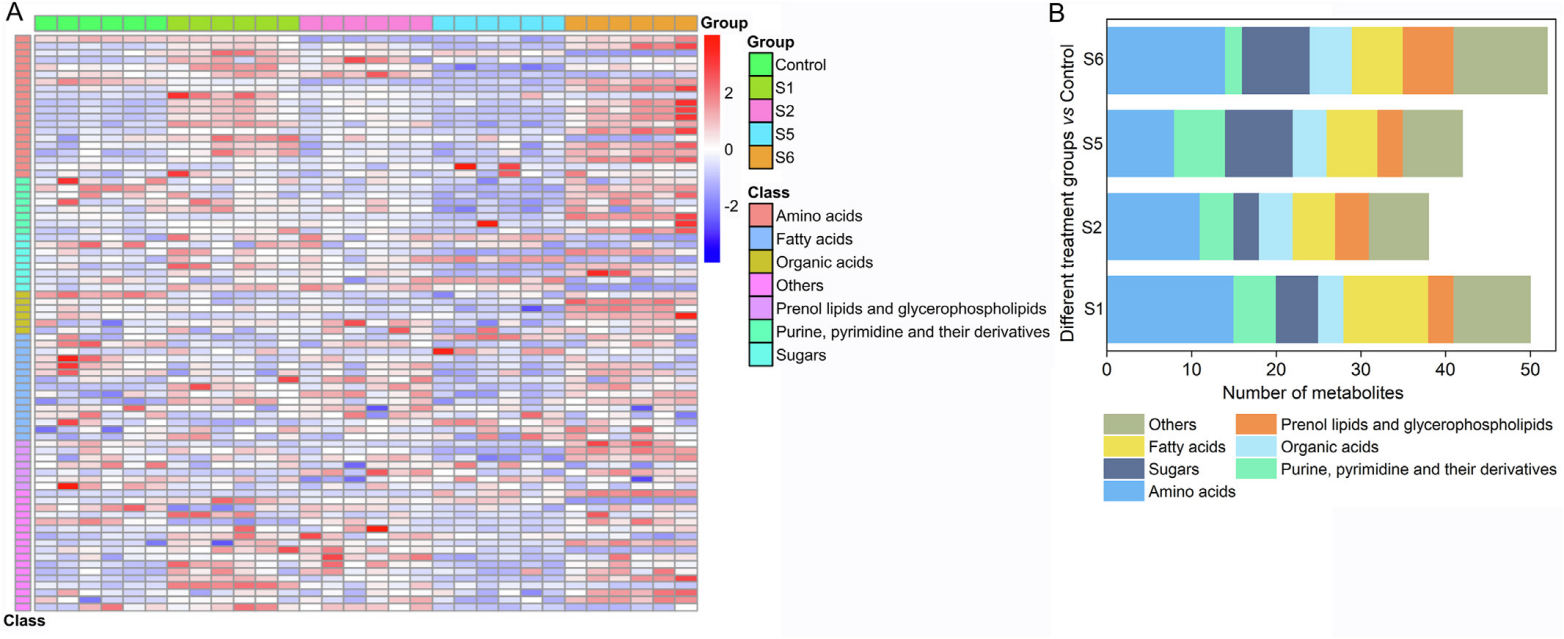
The significantly changed metabolites in ryegrass leaves exposure to soils contaminated by heavy metals showed by heat map (A) and the number of different classes of significantly regulated metabolites in treatment groups compared to the control group (B).

To visualize the overall changes in metabolites between control and groups with different OP values, PCA analysis of all detected metabolites was performed. Along the first principal axis, all treated groups were clearly separated from the control (Fig. S4). In addition, according to the volcano plots (Fig. S5), a total of 345 metabolites were detected. Compared to the control group, 31, 26, 11, and 38 metabolites were significantly up-regulated, and 19, 12, 31, and 14 metabolites were significantly down-regulated in the S1, S2, S5, and S6 treatments, respectively (Fig. S5). There were 50, 38, 42, and 52 significant differentially expressed metabolites in the S1, S2, S5, and S6 treatments, with a total of 81 significant differentially expressed metabolites based on *p* ​< ​0.05 and VIP >1 (Fig. 2). The relative contents of all 81 metabolites is shown in Fig. 2A. This indicates that the metabolite profiles of ryegrass leaves significantly changed after exposure to soils with different OP values. Plants are sensitive to stimuli in environmental conditions, which can lead to the imbalance of redox state [52]. Excessive ROS in plants can affect the cellular and molecular functions of plants and cause changes in the metabolites [52,53].

Significant changes between the treatments and the control group were observed in seven classes of metabolites, including amino acids, purines, pyrimidines and their derivatives, sugars, organic acids, fatty acids, prenol lipids, and glycerophospholipids, as well as other metabolites (Fig. 2B). The number of amino acids was the highest among metabolites in different treatment groups (Fig. 2B). In addition, compared to that in the control group, most of the amino acid metabolites such as l-proline, l-phenylalanine, and l-serine, were up-regulated in the treatments (Fig. 2A), which may indicate that the amino acid synthesis of ryegrass was promoted after exposure to soils with higher OP values, thereby improving the stress resistance. Amino acids play an important role in plant resistance to oxidative stress. For example, proline can directly scavenge free radicals and affect the transcription levels of antioxidant enzyme genes, thereby inducing oxidative stress, while other amino acids can indirectly act as signaling molecules involved in plant development and antioxidant stress [54].

In addition to amino acid metabolites, other metabolites, such as fatty acids, organic acids, and sugars, have significantly changed. Compared to the control group, sugar metabolites, such as l-sorbose, trehalose, and sucrose, were upregulated in the S1 and S2 treatments, which may be due to the fact that sugars may induce stress response signals and enhance plant resistance to stress by directly acting as negative signals or altering cellular response pathways [55]. The contents of some sugars, such as d-mannose and melibiose, decreased in the S5 and S6 treatments, which may be related to the fact that soils with higher OP values reduce sugar contents by regulating hormone signal transduction. Most of the organic acids and fatty acids were down-regulated in the S1, S2, and S5 treatments, and up-regulated in the S6 treatment. Different contamination levels can result in different regulation of metabolites, which may be related to plant self-regulation [56,57]. ROS can cause disruption of energy metabolism and redox balance, lipid peroxidation, nucleic acid damage, etc. These metabolites were involved in the maintenance of redox balance, synthesis of metabolites with ROS scavenging capacities, generation and consumption of ATP, and stabilization and protection of cell membranes in plants, all of which were associated with oxidative stress [58].

#### 3.3.2 Relationship between metabolic effect level index and OP values

**Fig. 3:**
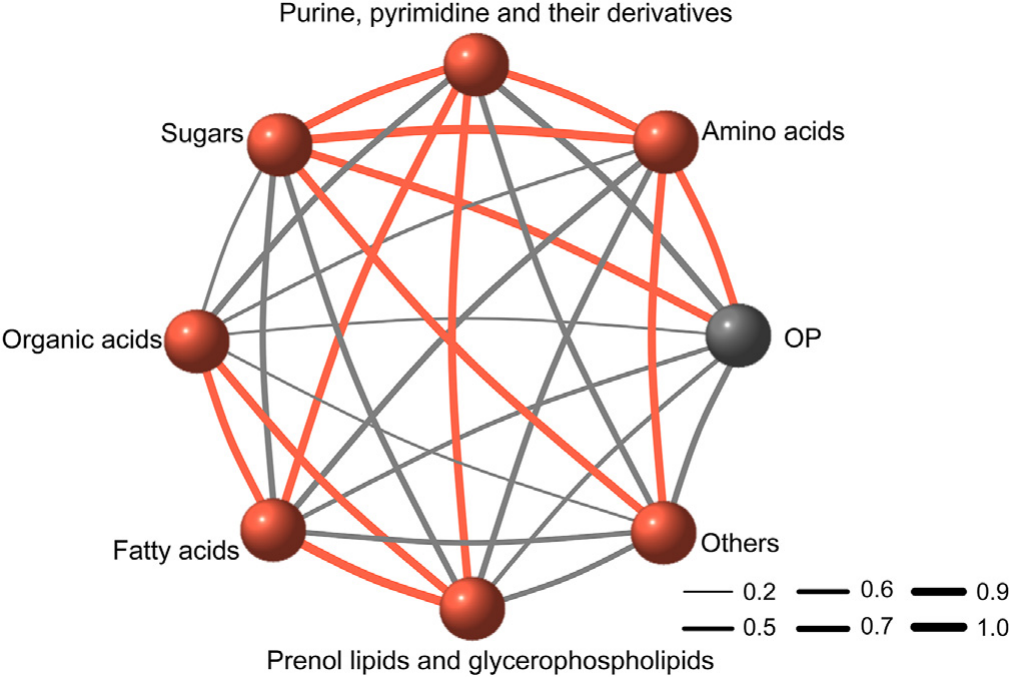
The correlation between OP value and MELI values of different classes of significantly regulated metabolites. The thickness of the line indicates the correlation coefficient. The red lines represent significant correlation with *p* ​< ​0.05 and the grey lines represent insignificant correlation with *p* ​> ​0.05.

To quantitatively reflect the aggregated metabolic changes in ryegrass with different soil OP, MELI of different classes of 81 metabolites were calculated, and the relationship between MELI and OP was evaluated. Compared to the control, the MELI values were significantly higher in the treatment groups (Fig. S6), which was consistent with the evident separation between control and treatment groups shown in the PCA analysis (Fig. S4), indicating that higher oxidative stress may induce more metabolic disturbance. The MELI of the lipophilic metabolites of *M. spicatum* significantly increased after two days of isoproterenol exposure due to oxidative stress [37]. A significant correlation between OP and MELI of amino acids and sugar metabolites in ryegrass was found (Fig. 3). There were also significant correlations among the MELI of other classes of metabolites (Fig. 3), indicating that OP was directly or indirectly related to the disturbance of metabolites in ryegrass leaves exposed to Cu and Pb-contaminated soils.

Plants under excessive ROS stress interact with other organelles through a “fast” signaling communication network and establish appropriate defense responses [59]. OP has been linked to the potential to induce ROS formation [60], and the OP values of fine particulate matter have been reported to mainly perturb amino acid metabolism, lipid metabolism, sugar biosynthesis, as well as cofactor and vitamin metabolism in the plasma of participants from two dormitories near and far from a congested highway [16]. The correlation between OP and MELI of specific metabolites, especially amino acids and sugars, in ryegrass was first reported in our study, illustrating the relationship between OP and metabolic disturbances of plant induced by oxidative stress.

### 3.4 Identification of important metabolites related to OP values by machine learning

To screen the most important metabolites related to OP values, different machine learning algorithms were used to rank the importance of metabolites. The performance of four machine learning models, including XGBoost, MLPClassifier, decision tree, and random forest, was optimized through the tuning of hyperparameters, and the optimized hyperparameter sets of the four models are shown in Table S4.

The best model was selected by cross-validated accuracy, recall, precision, F1 score, ROC, and AUC [61]. The F1 score achieved by the four models was 0.80–0.97 (Table S5), indicating a good balance between precision and recall. Random forest yielded the highest cross-validated accuracy (97.0%), while the cross-validated accuracies of the XGBoost and decision tree models were 77.0% and 60.0%, respectively (Table S5). The optimization did not converge when maximum iterations (200) were reached through five-fold cross validation with the MLPClassifier model (Table S5). Moreover, ROC curves of the four machine learning methods further illustrated that random forest performed best (Fig. S7). AUC values of random forest reached 1. Therefore, the random forest model was selected to rank the significantly differential metabolites based on their importance to OP values.

**Fig. 4:**
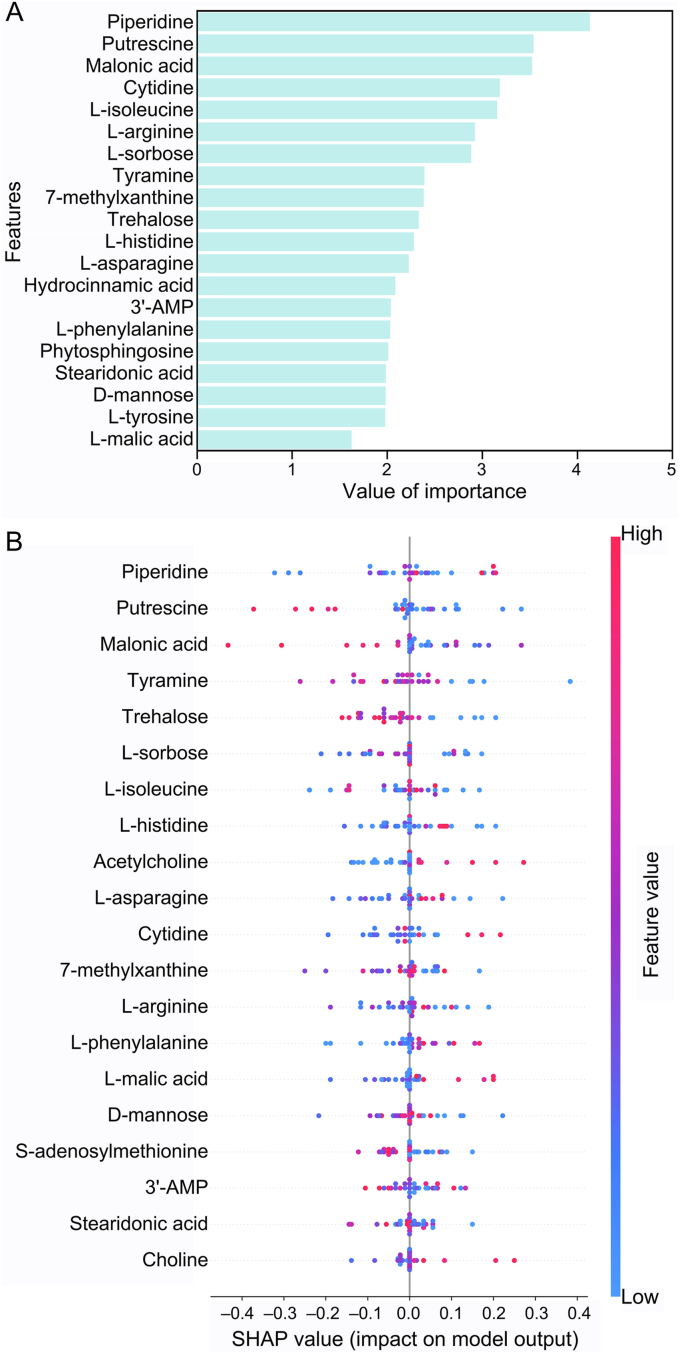
The importance ranking of the top 20 metabolites related to the OP values based on the random forest model (A) and the SHAP global explanation of top 20 features (B). The horizontal position represents the SHAP value of each data point, and the color reflects the content of the features. The point less than zero SHAP value represents a negative contribution to the output, while the point on the right represents a positive contribution.

Eighty-one significant differentially expressed metabolites between the treatment groups and the control group were involved in the analysis and ranked by the feature's importance. The relative abundances of the top 20 metabolites are shown in Fig. S8, and their sum importance to OP arrived 50.6% (Fig. 4A). The top 20 metabolites with the highest importance can be divided into six categories, including seven amino acids (i.e., l-isoleucine, l-arginine, tyramine, l-histidine, l-asparagine, l-phenylalanine, l-tyrosine), three purine, pyrimidine and their derivatives (i.e., cytidine, 7-methylxanthine, 3′-AMP), two organic acids (i.e., malonic acid, l-malic acid), three sugars (i.e., trehalose, l-sorbose, d-mannose), one fatty acid (i.e., stearidonic acid) and four other metabolites (i.e., piperidine, putrescine, hydrocinnamic acid, phytosphingosine).

Feature importance can be assessed based on random forest algorithm. To further intuitively explain the contribution of these features to the model, SHAP was used for in-depth model analysis [62]. SHAP values of the top 20 features contribution obtained from SHAP analysis for each data point are shown in Fig. 4B. Seventeen of the top 20 metabolites screened based on SHAP analysis were the same as the rank results of random forest, which further verified the correctness of the rank results of random forest. The SHAP explanation provided a more comprehensive understanding of the relationships and interactions within the dataset [27]. In the global SHAP explanation, the most important feature is “piperidine”, which is consistent with the random forest results. Some metabolites, such as l-malic acid and l-phenylalanine, have larger contents with higher SHAP values, indicating that these metabolites have a positive correlation with OP values, while some metabolites such as trehalose and putrescine showed a negative correlation with OP values (Fig. 4B). The top 20 most important metabolites for OP were identified through machine learning (Fig. 4A), providing further indication for the relationship between OP and metabolic disturbances of plants.

### 3.5 The identified metabolic pathways related to oxidative stress

**Fig. 5:**
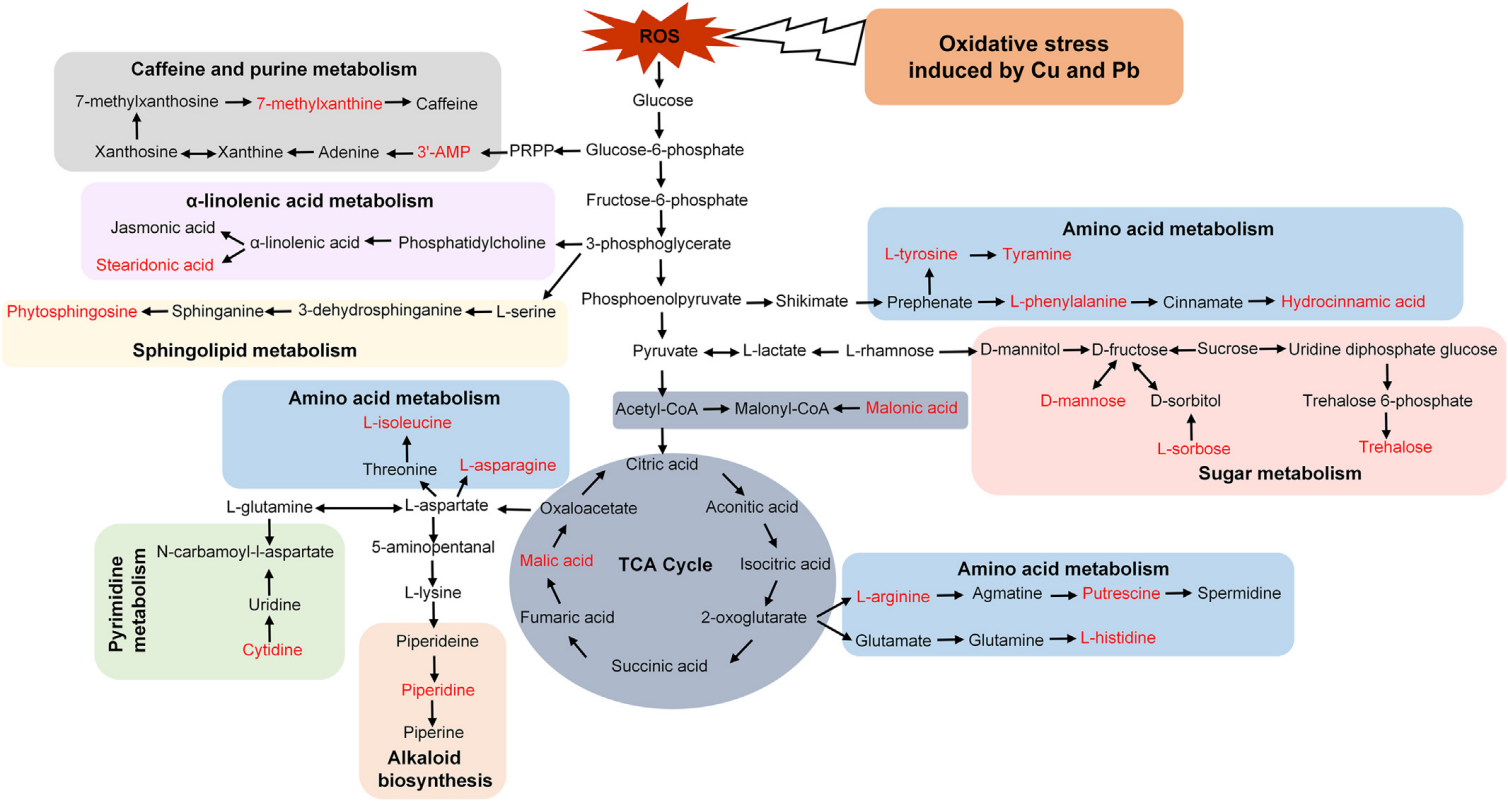
Schematic diagram of the proposed metabolic pathway of ryegrass related to OP values after exposed to Cu and Pb-contaminated soils. Red indicates the top 20 important metabolites related to OP values identified based on the random forest model.

Based on the pathway enrichment analysis using the public database Kyoto Encyclopedia of Genes and Genomes (KEGG), the metabolic pathways of the top 20 metabolites most related to OP were analyzed by pathway enrichment analysis (Fig. 5), including amino acid metabolism, tricarboxylic acid (TCA) cycle, alkaloid biosynthesis, caffeine metabolism, purine and pyrimidine metabolism, sphingolipid metabolism, sugar metabolism, and α-linolenic acid metabolism.

ROS can affect alkaloid biosynthesis, and alkaloids such as piperine have a feedback regulatory effect on ROS, participating in the clearance of free radicals and ROS in plants to decrease oxidative stress (Fig. 5) [63,64]. Amino acid metabolic pathways are influenced by ROS, as ROS can form stable oxidative covalent modifications with histidine, arginine, lysine, and tyrosine, leading to the formation of carbonyl compounds of these amino acids (Fig. 5) [65]. The TCA cycle plays a crucial role in plant stress responses to heavy metals by supplying reductants and adenosine triphosphate (ATP) for various adaptive mechanisms such as ROS detoxification and the electron transport chain [66,67]. Purine, pyrimidine metabolism and ROS interact with each other, and purine, pyrimidine metabolism may lead to secondary ROS generation, exacerbating oxidative stress [68]. Caffeine biosynthesis is important for maintaining the redox state of plants, and caffeine plays an important role in maintaining the redox homeostasis of peppermint leaves during later stages of phenanthrene stress [69]. Sugar metabolism, involved with osmoregulatory substances such as d-mannose and trehalose, can affect the antioxidant defense of plants by regulating the expression levels of enzymatic antioxidants [[70], [71], [72]]. α-linolenic acid functions as an antioxidant in plant lipid metabolism and serves as a precursor for the production of jasmonic acid, a recognized signaling molecule involved in subsequent antistress responses [73,74]. Sphingolipid metabolism can be affected due to the damage to the fluidity of membranes by excessive ROS [75]. Overall, the above metabolites were directly or indirectly related to oxidative stress induced by heavy metals.

## 4 Conclusions

OP of the *in situ* Cu and Pb contaminated soils were measured by DTT method, and biomarkers related to oxidative stress were determined. An exponential correlation between soil OP and the biomarker response index of ryegrass was observed. OP was found to be significantly associated with MELI of amino acids and sugars. By using random forest machine learning, the top 20 metabolites in plants and metabolic pathways related to OP values were identified. SHAP analysis was used to interpret the feature's contribution and the influence direction of the model. Metabolic pathways, such as amino acid metabolism, caffeine and purine metabolism, α-linolenic acid metabolism, sphingolipid metabolism, pyrimidine metabolism, alkaloid biosynthesis, TCA cycle, and sugar metabolism, were identified to be related to OP values. As a whole, OP can be used to evaluate the oxidative stress in plants induced by heavy metal-contaminated soil. The applicability of OP values in the ecological risk assessment of heavy metals or other contaminants on more types of plants can be further studied in the future.

## CRediT authorship contribution statement

**Chunmei Ran:** Writing – review & editing, Writing – original draft, Validation, Investigation. **Meiqi Guo:** Validation, Investigation. **Yuan Wang:** Validation, Formal analysis. **Ye Li:** Validation, Formal analysis. **Jiao Wang:** Writing – review & editing, Validation. **Yinqing Zhang:** Validation, Writing – review & editing. **Chunguang Liu:** Writing – review & editing, Supervision. **Bridget A. Bergquist:** Writing – review & editing, Supervision. **Chu Peng:** Writing – review & editing, Supervision, Methodology.

## Declaration of competing interests

The authors declare that they have no known competing financial interests or personal relationships that could have appeared to influence the work reported in this paper.
