# Supplementary material for: Can oxidative potential be a plant risk indicator for heavy metals contaminated soil? Analysis of ryegrass (Lolium perenne L.) metabolome based on machine learning
Source: Eco Environ Health. 2025 Mar 3;4(2):100140. doi: 10.1016/j.eehl.2025.100140 (PMC12002993; doi:10.1016/j.eehl.2025.100140)
Supplement: Multimedia component 1 [file mmc1.docx]

**SUPPORTING INFORMATION**

**Can oxidative potential be a plant risk indicator for heavy metals contaminated soil? Analysis of ryegrass (*Lolium perenne L.*) metabolome based on machine learning**

Chunmei Ran ^a,b^, Meiqi Guo ^b^, Yuan Wang ^b^, Ye Li ^b^, Jiao Wang ^c^, Yinqing Zhang ^b^, Chunguang Liu ^b^, Bridget A. Bergquist ^a^, Chu Peng ^b,^*

^a^ Department of Earth Sciences, University of Toronto, Toronto, Ontario M5S 3B1, Canada.

^b^ MOE Key Laboratory of Pollution Processes and Environmental Criteria, College of Environmental Science and Engineering, Nankai University, Tianjin 300071, China

^c^ School of Energy and Environmental Engineering, Hebei University of Technology, Tianjin 300401, China

*Number of pages: 18*

*Number of texts: 3*

*Number of figures: 8*

*Number of tables:5*

**Text S1.** Brief introduction to four machine learning models

Four commonly used classification models were tested to find the most suitable machine learning model for the data in this study. These models are fully supervised machine learning classification models, including nonlinear and integrated ensemble methods.

**1. Random forest**

Random forest is a forest constructed in a random manner with many decision trees and each decision tree is independent. The average of the results of all training trees on a random subset of the same data is used as the final prediction result. Random forests can reduce overfitting and prediction variance [1].

**2. eXtreme gradient boosting (XGboost)**

XGboost is a type of synthetic algorithm that combines basis functions and weights to form a better fitting effect on data. XGboost and GBDT belong to boosting algorithms, while XGboost has improved the traditional GBDT algorithm by adding regularization terms to the loss function to control model complexity, allowing the objective function to consider both loss function and structural risk. The entire algorithm is moving towards minimizing the objective function [2].

**3. Decision tree**

A decision tree is a tree structure in which each leaf node represents a category, each internal node represents a test on an attribute, and each branch represents a test output. Trees explain the changes of an individual response variable by repeatedly dividing data into more uniform groups, with each group characterized by the typical value of the response variable, the number of observations in the group, and the value of the explanatory variable that defines it [3].

**4. Multi-layer perceptron classifier (MLPClassifier)**

MLPClassifier is an artificial neural network algorithm based on the principle of backpropagation algorithm, which minimizes the loss function by continuously adjusting weights and thresholds. The backpropagation algorithm first passes the input signal from the input layer to the output layer through forward propagation, and calculates the model's prediction results. Then, by calculating the gradient of the loss function, the algorithm can determine the contribution of each neuron to the loss function, and adjust weights and thresholds based on these contributions. This process is repeated multiple times until the difference between the predicted results of the model and the actual values is minimized [4].

**Text S2.** The calculation of cross-validation accuracy, recall, precision and F1 score

$$Recall= \frac{TP}{TP+FN}$$

$$Precision= \frac{TP}{TP+FP}$$

$$F1= \frac{2\times recall \times precision}{recall+precision}=\frac{2\times TP}{2\times TP+FP+FN}$$

$$Accuracy= \frac{TP+TN}{TP+TN+FP+FN}$$

$$CV Accuracy=\frac{1}{k}\sum_{i=1}^{k} {Accuracy}_{i}$$

where *TP* is the true positive prediction, *TN* is the true negative prediction, *FP* is the false positive prediction, and FN is the false negative prediction. *Accuracy_i_* is the accuracy of the *i*-th fold in cross-validation, and *k* is the number of folds [5].

**Text S3.** Brief introduction to SHapley Additive exPlanations (SHAP)

SHAP explains the output of machine learning models based on the Shapley value, which is a method for allocating benefits to participants in coalitional game theory. The principle of SHAP is to quantify the contribution of each feature as its increase or decrease in the model output, and consider the impact of each feature as its average marginal contribution under all possible feature combinations. SHAP satisfies three desirable properties of local accuracy, missing values, and consistency, while finding a unique solution for coalitional game theory [6-8].


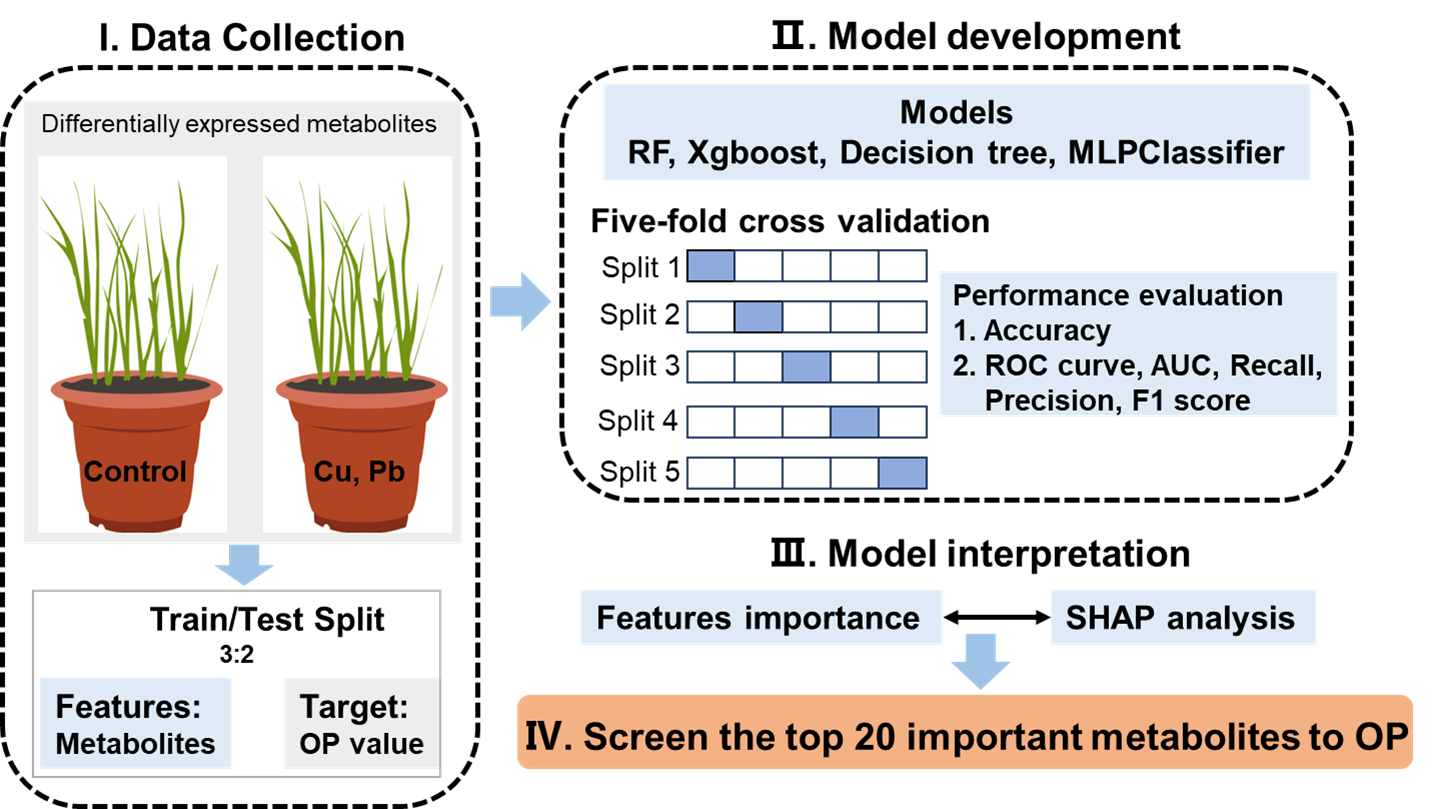


**Fig. S1** Workflow of the machine learning analysis.

**Fig. S2** ROS in the ryegrass exposed to the industrial contaminated soils and the control soil. (FW: fresh weight of ryegrass)

**Fig. S3** CAT, T-AOC and MDA (A); SOD, POD and MT (B) in the ryegrass exposed to the industrial contaminated soils and the control soil.


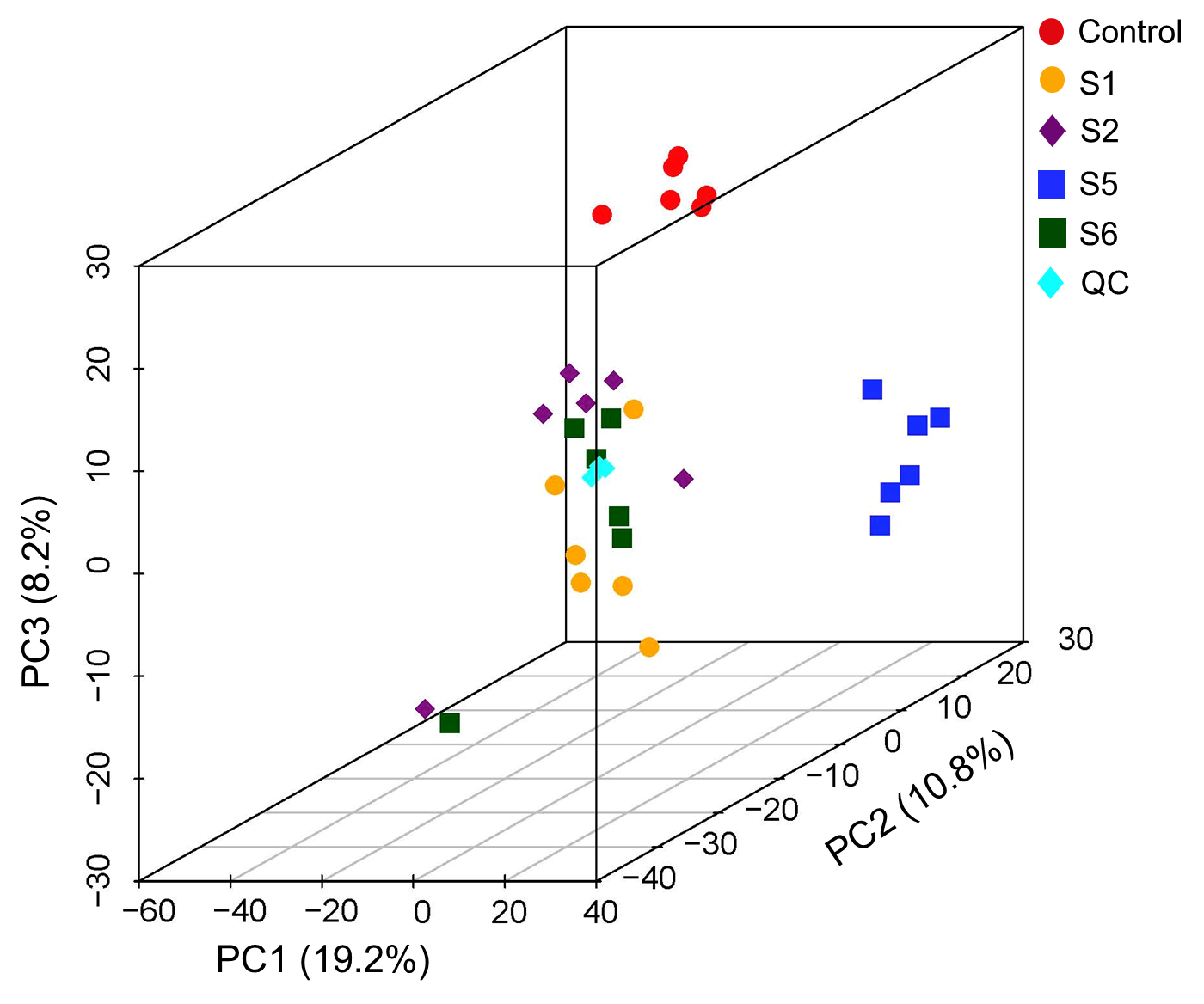


**Fig. S4** Score plot for PCA model total with QC of the metabolomics in ryegrass planted in different heavy metal contaminated soils.

QC: quality control; QC samples located at the center and more clustered indicate better method stability and higher data quality.


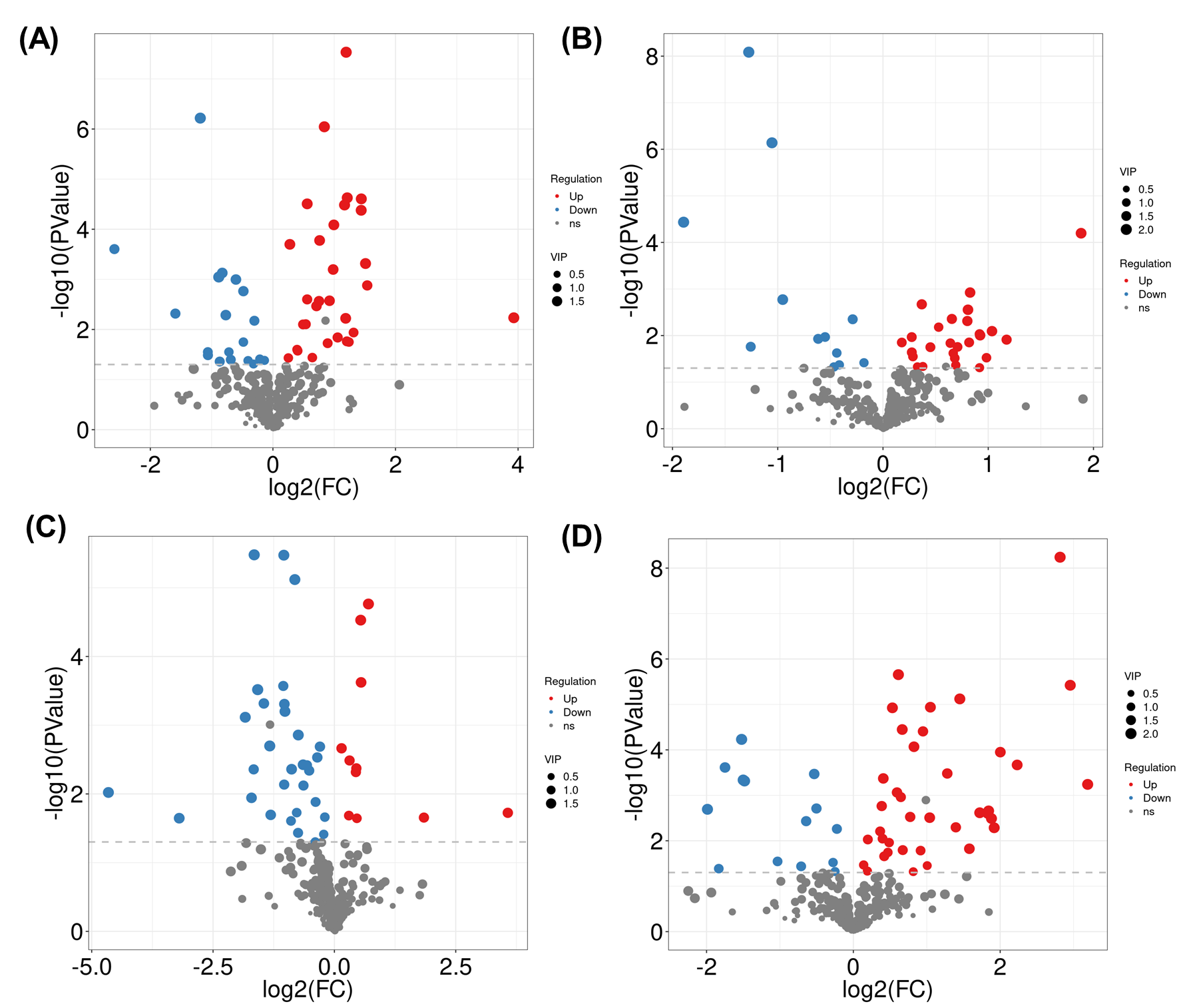


**Fig. S5** Molecular feature volcano plots of the group of ryegrasses exposed to “the S1 soil” *vs*. “Control” (A) and “the S2 soil” *vs.* “Control” (B), and “the S5 soil” *vs.* “Control” (C) and “the S6 soil” *vs.* “Control” (D).

Each point represents a feature. The blue and red dots represent the fold changes smaller than 1 and larger than 1, while the *p* < 0.05 and VIP > 1.

**Fig. S6** MELI values of different classes of metabolites.


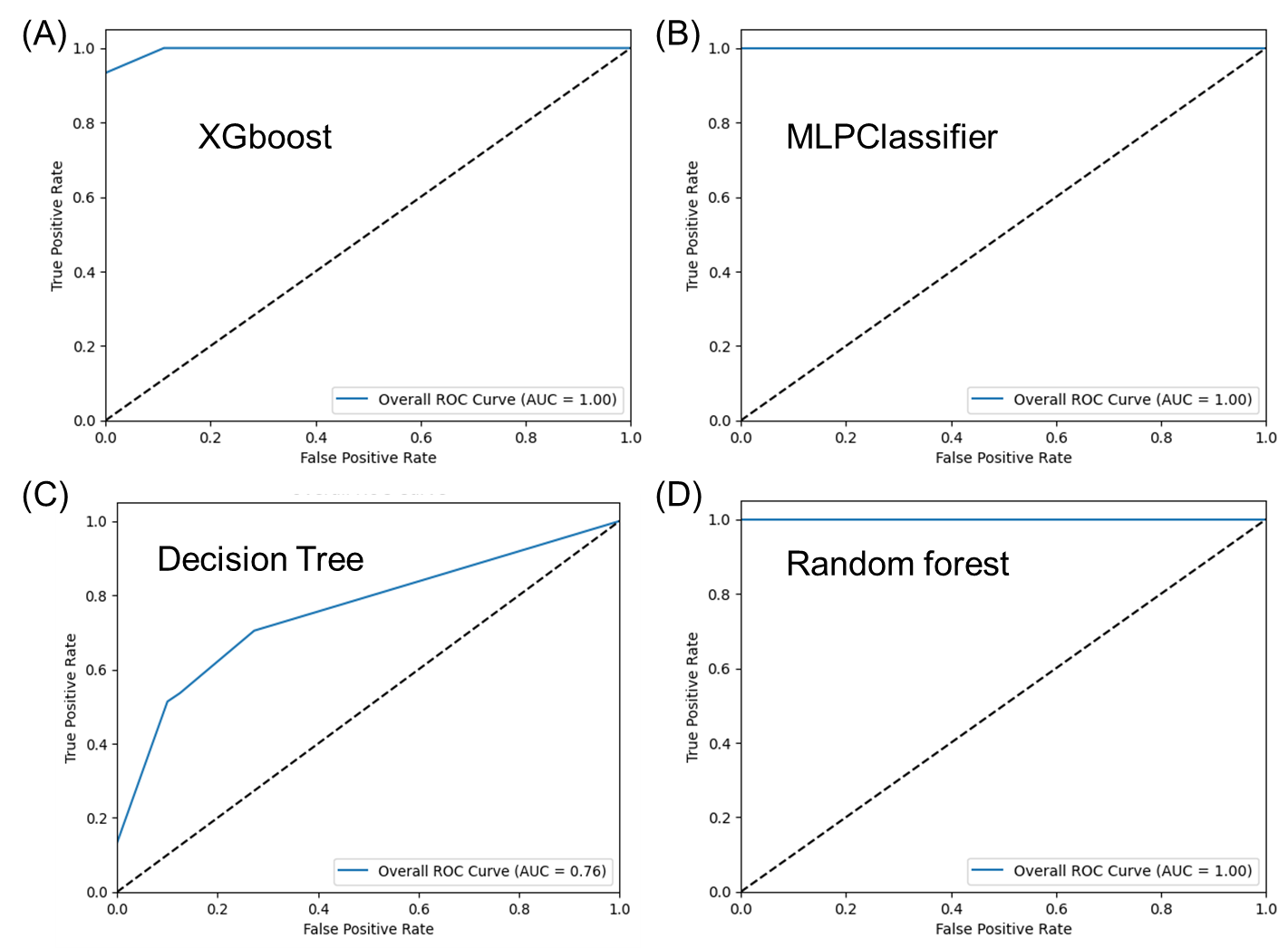


**Fig. S7** Performance evaluation of different machine learning model using the area under the curve (AUC) value and receiver operating characteristic (ROC) curve based on the significantly differential metabolites dataset, including XGboost, MLPClassifier, decision tree and random forest.


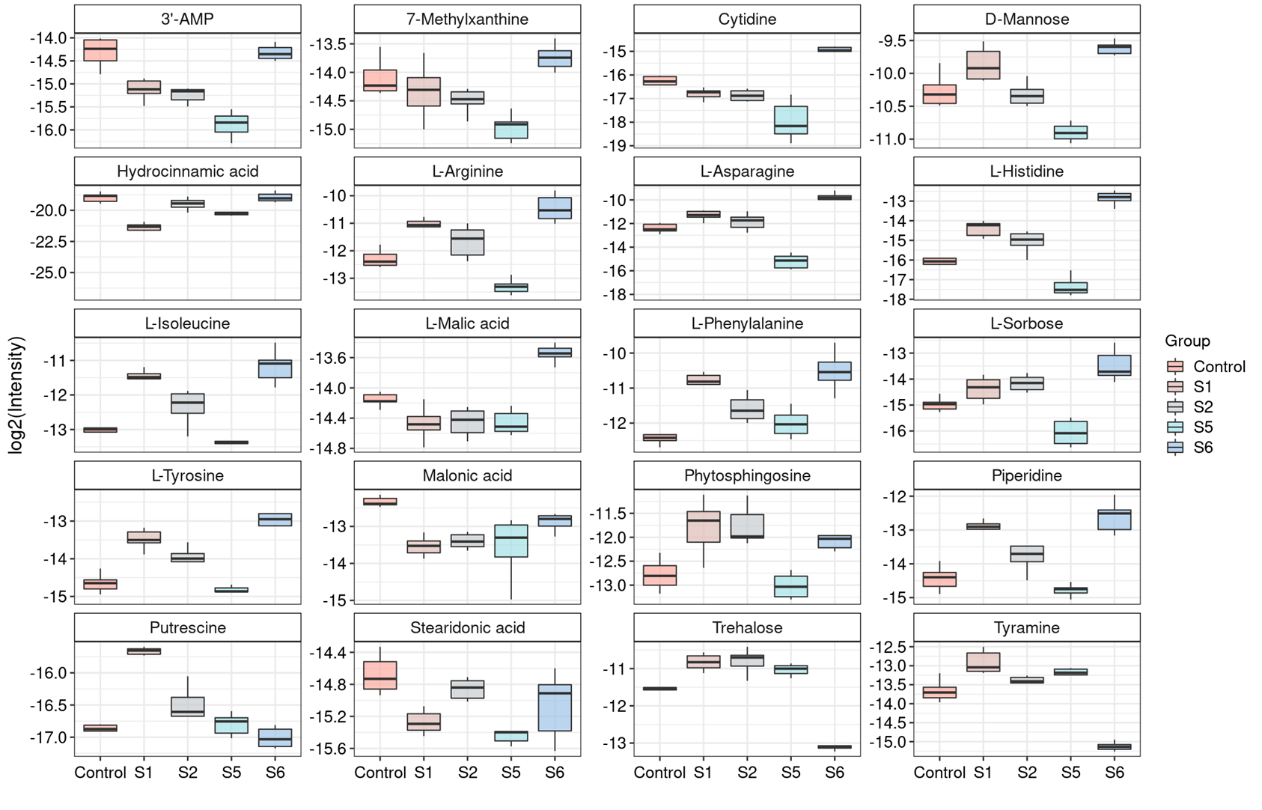


**Fig. S8** The relative abundance of the selected top 20 metabolites based on the random forest model.

**Table S1** Cu and Pb concentrations in the soils and the water extracts of soils samples and the OP values of soils

|  | Cu  (mg/kg) | Pb  (mg/kg) | Cu  (μM) | Pb  (μM) | OP  [pmol/(min·μg)] |
| --- | --- | --- | --- | --- | --- |
| Control | 21.5±1.34 | 10.4±0.95 | 0.0783 | - | 20.9±1.11 |
| S1 | 189± 12.1 | 78.0±20.1 | 0.155 | 0.00521 | 25.5±2.33 |
| S2 | 491±32.4 | 195±45.4 | 0.313 | 0.00558 | 31.2±4.31 |
| S3 | 891±49.6 | 385±50.4 | 0.637 | 0.00987 | 32.3±3.37 |
| S4 | 1562±188 | 90.3±12.1 | 0.755 | 0.00399 | 35.5±4.15 |
| S5 | 2004±54.7 | 702±36.6 | 0.951 | 0.0192 | 45.5±1.49 |
| S6 | 6909±86.6 | 161±6.86 | 1.25 | 0.00697 | 52.6±2.05 |
| S7 | 392±31.5 | 543±35.7 | 1.47 | 0.384 | 59.2±3.22 |
| S8 | 671±41.1 | 629±16.8 | 1.93 | 0.252 | 62.8±4.26 |

-: The concentration of Pb was lower than the limit of detection.

**Table** **S2** Physical and chemical properties of soils

| **Soil samples** | **pH** | **SOC**^a^ **(mg/g)** | **C/N** | **Texture** |
| --- | --- | --- | --- | --- |
| Control | 6.85 | 18.9±2.92 | 17.3±2.77 | Loam |
| S1 | 6.92 | 19.0±2.15 | 19.7±3.41 | Loam |
| S2 | 7.25 | 15.7±1.26 | 17.6±3.15 | Loam |
| S3 | 7.34 | 17.9±1.87 | 16.3±1.98 | Loam |
| S4 | 7.10 | 16.6±3.30 | 22.8±4.25 | Loam |
| S5 | 7.27 | 18.8±2.91 | 26.9±5.21 | Loam |
| S6 | 7.47 | 16.7±3.57 | 25.0±3.08 | Loam |
| S7 | 7.36 | 19.4±2.10 | 27.2±2.09 | Loam |
| S8 | 7.17 | 15.3±1.62 | 21.8±3.85 | Loam |

^a^ Soil organic carbon.

**Table S3** Parameters of LC gradient elution program and mass detection

| Contents | Parameters |
| --- | --- |
| Mobile Phases | A. 5 mmol/L ammonium acetate in water  B. 5 mmol/L acetic acid in acetonitrile |
| Gradient Profile | 0–1 min: 2% B;  1-19 min: 2% B–100% B;  19–21 min: 100% B;  21–21.1 min: 100% B–22% B;  21.1–25 min: 2% B. |
| Flow rate | 0.30 mL/min |
| MS | Full scan mode  Resolution = 60000  Scan range (*m/z*) = 70–1000  Spray voltage (kV) = 3.8 (positive ionization mode)  3.4 (negative ionization mode)  Sheath gas flow rate (arbitrary units) = 50  Auxiliary gas flow rate (arbitrary units) = 15  Auxiliary gas heater temperature (°C) = 320  Capillary temperature (°C) = 320 |
| MS/MS | Higher-energy collisional dissociation (HCD) mode  Collision energy (eV) = 10, 30, 60 |

**Table S4** Summary of hyperparameter optimization

| Indice/Hyperparameter | Random forest | XGboost | Decision tree | MLPClassifier |
| --- | --- | --- | --- | --- |
| Max depth | 5^a^ | 3 | 4 | - |
| n_Estimators | 100 | 100 | - | - |
| Alpha | - | - | - | 0.0001 |
| Minimum samples leaf | 1 | - | 1 | - |
| Minimum sample split | 2 | - | 2 | - |
| Learning rate | - | 0.2 | - | - |
| Criterion | - | - | Entropy | - |
| learning_rate_init | - | - | - | 0.001 |
| Sizes of hidden layers | - | - | - | 100 |
| Max _features | - | - | Sqrt | - |
| Activation function | - | - | - | Relu |
| Solver | - | - | - | Adam |

^a^: The parameters were optimized using a grid search method;

-: The parameters were default values.

**Table S5** Comparison of the matrices for evaluating model performances in five-fold cross validation

| Model | Cross-validated Accuracy | Precision | Recall | F1-score |
| --- | --- | --- | --- | --- |
| XGboost | 77.0% ± 40.0% | 0.95 | 0.933 | 0.93 |
| MLPClassifier | -- | 0.971 | 0.967 | 0.97 |
| Decision tree | 60.0% ± 39.0% | 0.805 | 0.80 | 0.80 |
| Random forest | 97.0% ± 13.0% | 0.871 | 0.867 | 0.87 |

--: maximum iterations (200) reached while the optimization hasn't converged yet.

**References**

[1] H. Tyralis, G. Papacharalampous, A. Langousis, A brief review of random forests for water scientists and practitioners and their recent history in water resources, Water 11 (2019) 910. https://doi.org/10.3390/w11050910.

[2] T. Chen, C. Guestrin, Xgboost: A scalable tree boosting system, in Proceedings of the 22nd acm sigkdd international conference on knowledge discovery and data mining, 2016, 785-794. https://doi.org/10.1145/2939672.2939785.

[3] G. De'ath, K. E. Fabricius, Classification and regression trees: a powerful yet simple technique for ecological data analysis, Ecology 81 (2000) 3178-3192.

https://doi.org/10.1890/0012-9658(2000)081[3178:CARTAP]2.0.CO;2.

[4] S. Wan, Y. Liang, Y. Zhang, M. Guizani, Deep multi-layer perceptron classifier for behavior analysis to estimate Parkinson’s disease severity using smartphones, IEEE Access 6 (2018) 36825-36833. https://doi.org/10.1109/ACCESS.2018.2851382.

[5] L. Xin, H. Yu, S. Liu, G. G. Ying, C. E. Chen, POPs identification using simple low-code machine learning, Science of The Total Environment, 921(2024) 171143. https://doi.org/10.1016/j.scitotenv.2024.171143.

[6] D. Meddage, I. U. Ekanayake, A. U. Weerasuriya, C. S. Lewangamage, K. T. Tse, T. P. Miyanawala, et al., Explainable machine learning (XML) to predict external wind pressure of a low-rise building in urban-like settings, Journal of Wind Engineering and Industrial Aerodynamics, 226 (2022) 105027. https://doi.org/10.1016/j.jweia.2022.105027.

[7] C. Madhushani, K. Dananjaya, I. Ekanayake, D. Meddage, K. Kantamaneni, U. Rathnayake, Modeling streamflow in non-gauged watersheds with sparse data considering physiographic, dynamic climate, and anthropogenic factors using explainable soft computing techniques, Journal of Hydrology, 631 (2024) 130846. https://doi.org/10.1016/j.jhydrol.2024.130846.

[8] I. Ekanayake, D. Meddage, U. Rathnayake, A novel approach to explain the black-box nature of machine learning in compressive strength predictions of concrete using Shapley additive explanations (SHAP), Case Studies in Construction Materials, 16 (2022) e01059. https://doi.org/10.1016/j.cscm.2022.e01059.
